# Real-World Clinical Effectiveness of Liraglutide for Weight Management in Türkiye: Insights from the LIRA-TR Study

**DOI:** 10.3390/jcm13206121

**Published:** 2024-10-14

**Authors:** Alihan Oral, Celalettin Küçük, Murat Köse

**Affiliations:** 1 Department of Internal Medicine, Faculty of Medicine, Biruni University, Gültepe, Halkalı Street Number: 99, 34295 İstanbul, Türkiye; celalettinkucuk@yahoo.com; 2 Department of Internal Medicine, Istanbul Faculty of Medicine, Istanbul University, 34093 İstanbul, Türkiye; murat.kose@istanbul.edu.tr

**Keywords:** obesity, liraglutide, weight loss

## Abstract

**Introduction:** Obesity is a complicated chronic disease associated with a series of other conditions. A weight loss of 5–10% has been shown to reduce obesity-related complications and improve quality of life. The efficacy and safety of liraglutide for reducing body weight have been demonstrated in clinical trials. This study evaluated the weight loss efficacy and adverse effects of liraglutide in those with obesity in the Turkish population. **Methods:** This is a retrospective cohort study; the patients that were included had a body mass index (BMI) of 27 or greater with additional comorbidities or a BMI of 30 or greater, and the patients were prescribed liraglutide for obesity treatment from the tertiary private clinic between January 2022 and January 2024. Their metabolic and anthropometric parameters were recorded at the initial appointment, and their body weight and adverse effects were followed up on during therapy. **Results:** For the 568 patients, of whom 487 (85.6%) were female, the mean values for age, weight, and BMI were 42.37 ± 10.50, 98.09 ± 17.48 kg, and 35.77 ± 5.45 kg/m^2^, respectively. Reductions in body weight at the 4th, 8th, 12th, and 24th weeks were 6.45 ± 2.32 kg, 10.66 ± 3.41 kg, 15.38 ± 8.30 kg, and 19 ± 9.06 kg, respectively; reductions in BMI at the 4th, 8th, 12th, and 24th weeks were 2.36 ± 1.00, 3.88 ± 1.25, 5.36 ± 1.76, and 7.09 ± 2.93, respectively; and the percentages of overall body weight loss at the 4th, 8th, 12th, and 24th weeks were 6.62 ± 2.1%, 10.75 ± 2.71%, 14.97 ± 6.8%, and 18.55 ± 4.63%, respectively (all *p* values < 0.0001). The percentage of patients who lost more than 5% and more than 10% of their initial weight was 100% at the 24th week. The most common side effect was nausea; no pancreatitis was observed. **Conclusions:** The results of our study indicate that liraglutide is an efficacious and safe treatment option for obesity in the Turkish population, in accordance with the findings from previous research.

## 1. Introduction

Obesity is a complicated chronic disease caused by an abnormal or excessive accumulation of fat in the body, which impairs health, increases the risk of long-term medical complications, and shortens life expectancy. Over the past three decades, the prevalence of obesity has steadily increased worldwide [1]. According to the World Health Organization’s (WHO’s) report on obesity in the WHO European region, almost 60% of adults are overweight or obese. With 66% of the population being overweight or obese, Turkey ranks first among European countries [1]. A weight loss of 5–10% has been shown to reduce obesity-related complications and improve quality of life, but weight loss is difficult to maintain with lifestyle interventions alone [2]. The implementation of lifestyle modifications, comprising a reduction in food intake and an increase in physical activity, has not resulted in consistent long-term weight loss due to physiological counter-regulatory mechanisms that are in operation [3,4]. According to the European Guidelines for the Management of Obesity in Adults, pharmacotherapy should be considered as part of a comprehensive disease management strategy and is recommended for adults with a body mass index (BMI) > 30 kg/m^2^ or a BMI > 27 kg/m^2^ with an obesity-related comorbidity [5]. Pharmacotherapy is a noninvasive approach that can help people with obesity adhere to weight loss strategies, improve their quality of life, and reduce the risk of comorbidities [6]. Indeed, pharmacological intervention, when used in conjunction with lifestyle modifications, is advised to facilitate the alleviation of obesity-related symptoms in adults who are overweight or obese and who are unable to achieve or sustain clinically significant weight loss through dietary and exercise regimens alone [7]. Liraglutide, an acylated glucagon-like peptide 1 (GLP-1) receptor agonist with a 97% homology to human GLP-1, is employed for the management of glycaemic control in patients with type 2 diabetes. However, in addition to this metabolic effect, this medication has also demonstrated efficacy as a regulator of appetite [8,9]. Liraglutide effectively suppresses appetite by acting on the arcuate nucleus of the hypothalamus, leading to a reduction in energy intake and subsequent weight loss [10]. It is indicated as an adjunct to diet and exercise for weight management in patients with a BMI of 27 or greater with additional comorbidities or a BMI of 30 or greater [10]. Liraglutide was approved by the US Food and Drug Administration (FDA) in December 2014 and by the European Medicines Agency (EMA) in 2015 for the treatment of obesity in combination with lifestyle interventions after multiple, large phase 3 randomised controlled trials demonstrated its safety and efficacy for weight loss, weight maintenance, and improvement in obesity-related complications [10,11,12,13]. The efficacy of 3.0 mg of liraglutide in a clinical context has been substantiated by randomised controlled trials. While these trials offered substantial insights into liraglutide’s safety and efficacy, the data from these trials were derived from highly controlled and specific populations; thus, the results may not be directly applicable to a real-world context. In a real-world setting, patients are subject to a greater number of uncontrolled variables and often experience a wider range of comorbidities and variable adherence to treatment, which can impact the efficacy of the drug in this context [14].

Utilising empirical evidence from real-world contexts, this study assesses the efficacy and safety of 3.0 mg liraglutide treatment in conjunction with dietary regimens and physical activity among a representative sample of patients from Türkiye.

## 2. Materials and Methods

### 2.1. Data Collection

We conducted this retrospective cohort study on patients with a BMI of 27 or greater with additional comorbidities or a BMI of 30 or greater who were prescribed liraglutide for obesity treatment between January 2022 and January 2024 at the Biruni University Hospital and Medicana Bahçelievler Hospital.

### 2.2. Patient Selection

**Figure 1:**
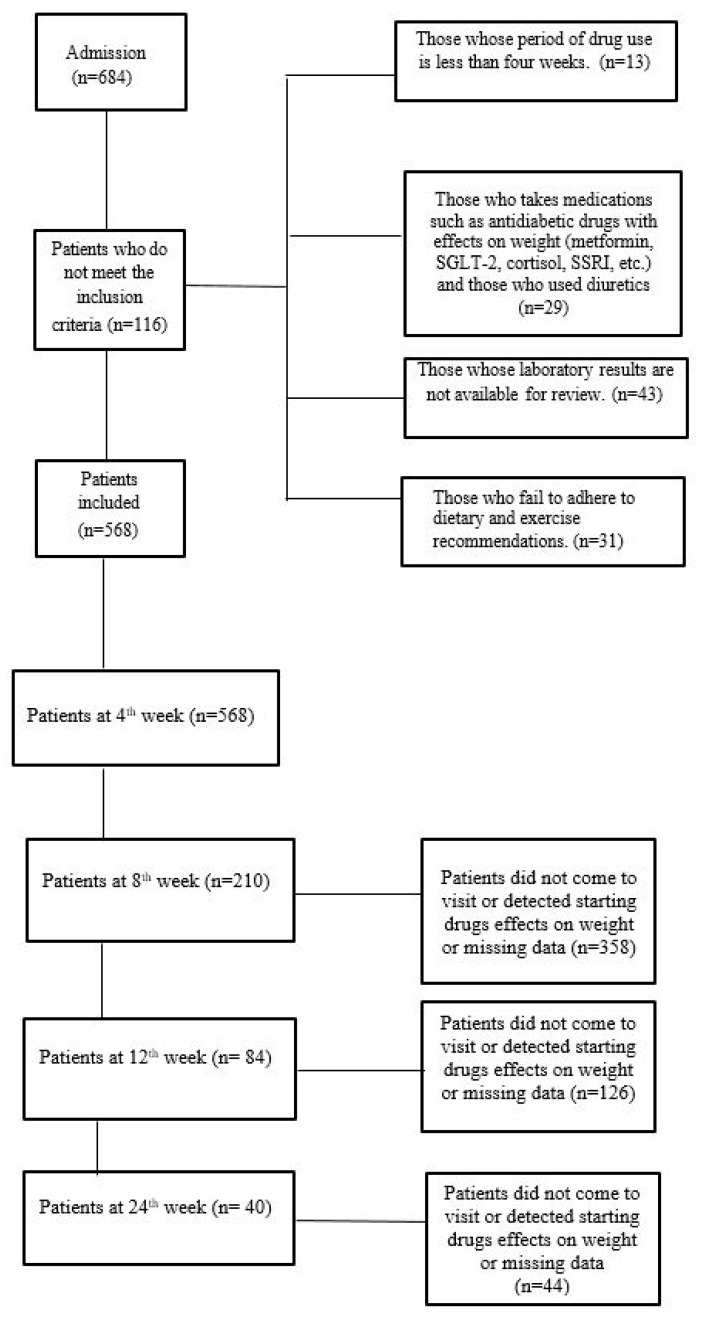
Flowchart of the number of patients included in the study. SGLT2, sodium–glucose cotransporter-2 inhibitors; SSRIs, selective serotonin reuptake inhibitors.

All patients were advised to adhere to a dietary regimen comprising 500 kilocalories per day less than their usual caloric intake and to engage in 150 min per week of physical activity. The data were retrospectively collected from the patients’ records. The exclusion criteria were as follows: age under 18; no baseline record (body weight and height) or no follow-up data for body weight; previous use of glucagon-like peptide (GLP-1) analogues or currently taking medications that affect weight, such as metformin, sodium–glucose cotransporter-2 (SGLT2) inhibitors, cortisol, selective serotonin reuptake inhibitors (SSRIs), and diuretics; previous bariatric surgery or conditions that could contribute to weight loss, such as a malignancy or chronic infection; and patients whose records showed that they were not following our diet and/or exercise recommendations (Figure 1). A diagnosis of diabetes or prediabetes was made in accordance with the criteria set forth by the American Diabetes Association (ADA) in 2023 or based on the use of antidiabetic drugs. A diagnosis of hypertension was based on a mean of three consecutive measurements above 140/90 mmHg, or the use of antihypertensive medication [15]. For enrolled patients, initial height, weight, BMI, age, sex, blood pressure values, transaminases, triglyceride, cholesterol, and glycated haemoglobin (HbA1c) data were collected at the initial exam. The Turkish Society of Endocrinology and Metabolism guideline recommends assessing weight change and drug side effects during the follow-up of obese patients [16]. For subsequent visits, body weight, BMI, and any adverse effects related to drug intake were recorded.

Dose escalation of liraglutide was made according to a phase 3 study of liraglutide, starting at a dose of 0.6 mg with weekly 0.6 mg increments to 3.0 mg [10].

All patients included in the study were monitored throughout the drug intake period. Throughout the treatment period, data regarding weight loss, waist circumference, and any adverse effects related to the drug were recorded at 4-, 8-, 12-, and 24-week follow-ups. At week 4, data from 568 patients were available; data from 210, 84, and 40 patients were available at weeks 8, 12, and 24, respectively. The weight loss achieved during this period was calculated continuously using the Tanita F1BC-601PRO (TANITA, Tokyo, Japan)at each clinic visit.

## 3. Statistics

Numerical data are provided as means and standard deviations. The frequency values are expressed as percentages. Given that the number of cases exceeded 30, a Kolmogorov–Smirnov test was employed to ascertain normality. The homogeneity of the data was evaluated using the Levene test. As the data were both normally distributed and homogeneous, a paired *t*-test was employed to assess weight and BMI changes at the 4th, 8th, 12th, and 24th weeks. Categorical data were calculated using the Chi-squared test. A generalised linear model for repeated measures (mixed ANOVA test) was employed to ascertain the impact of confounding factors on weight loss. Statistical analyses were performed using SPSS 25.00 (Chicago, IL, USA), and a 95% confidence interval was accepted. *p* < 0.05 was considered statistically significant.

## 4. Results

**Figure 2:**
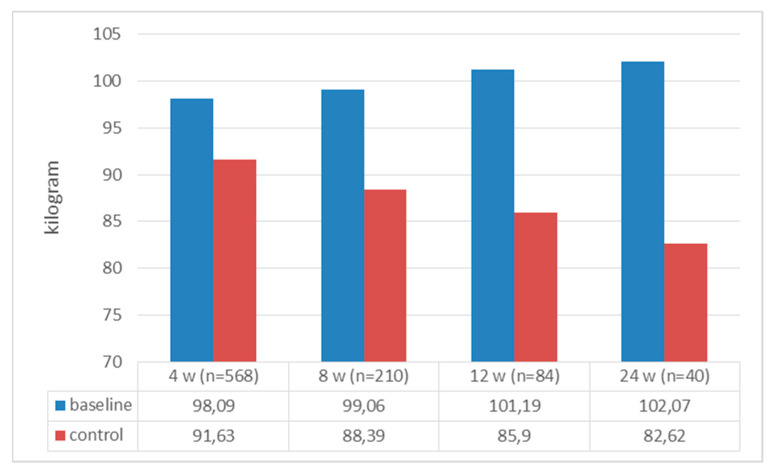
Body weight at initial appointment and at follow-up.

**Figure 3:**
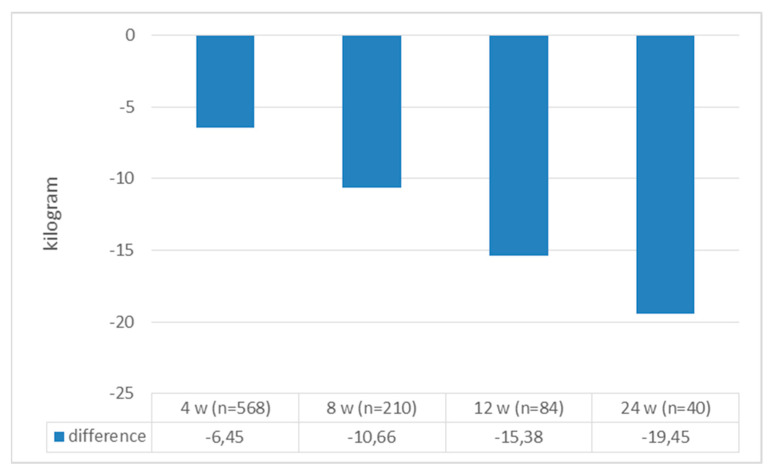
Body weight change at initial appointment and at follow-up.

**Figure 4:**
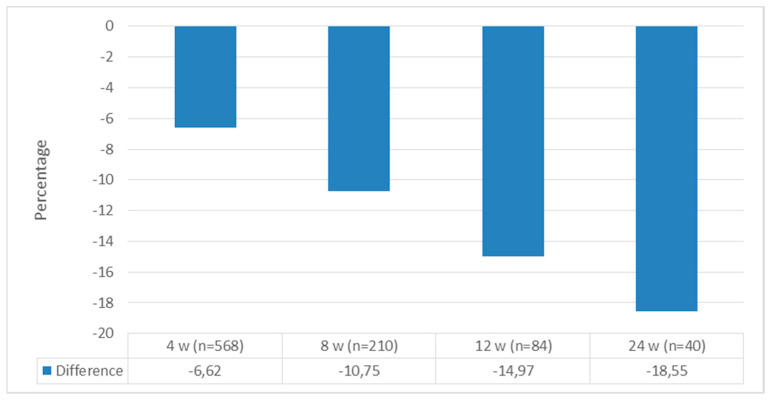
Body weight change compared to initial weight.

**Figure 5:**
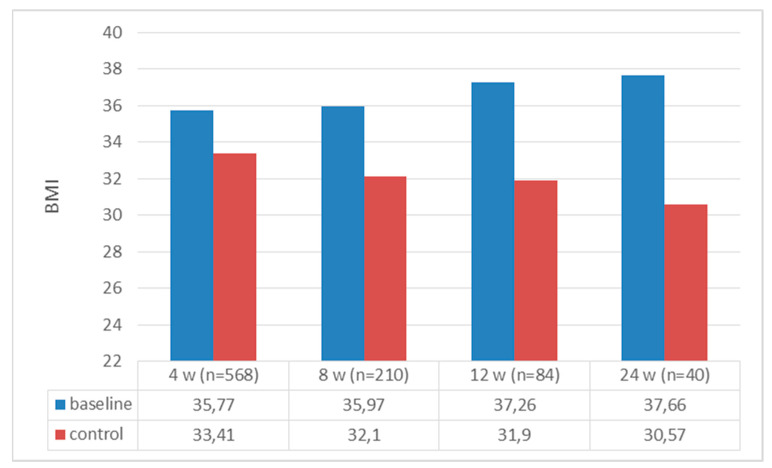
Body mass index at baseline and at follow-up.

**Figure 6:**
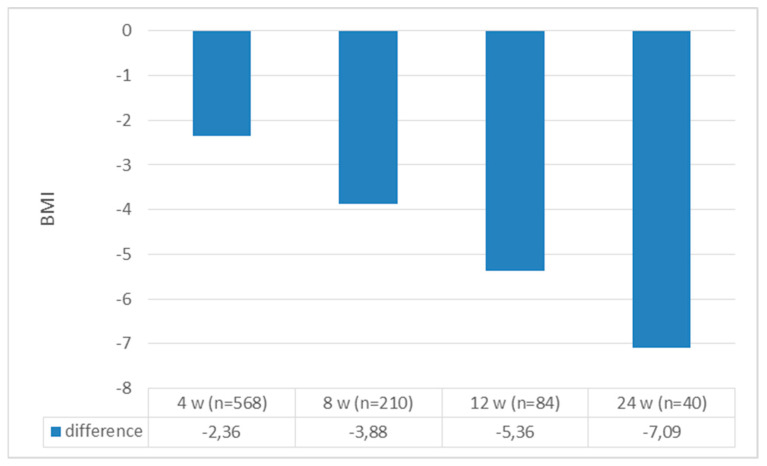
Change in BMI at follow-up.

A total of 568 patients were included in the study; 487 (85.6%) were female, with mean age, weight, and BMI values of 42.37 ± 10.50, 98.09 ± 17.48 kg, and 35.77 ± 5.45 kg/m^2^, respectively. Of those with chronic diseases, prediabetes was present in 37.9% of patients, DM was present in 7.8%, and hypertension was present in 64.7%. The baseline and follow-up baseline values for the patients are presented in Table 1 and Table 2, respectively. When we compared the patients’ initial weight with their weight at the 4th, 8th, 12th, and 24th weeks, all the weights at follow-up were significantly lower [4th week: 98.09 ± 17.48 to 91.63 ± 16.66, t(568) = 66,278 (*p* < 0.0001); 8th week: 99.06 ± 17.76 to 88.39 ± 15.92 t(210) = 45,233 (*p* < 0.0001); 12th week: 101.19 ± 20.57 to 85.90 ± 18.19 t(84) = 16,868 (*p* < 0.0001); and 24th week: 102.07 ± 23.17 to 82.62 ± 15.95 t(40) = 13,572 (*p* < 0.0001)] (Figure 2). When we looked at how much weight the patients lost compared to their initial weight, all the weight differences were statistically significant [6.45 ± 2.32 kg, t(568) = 66,278 (*p* < 0.0001); 10.66 ± 3.41 kg, t(210) = 45,233 (*p* < 0.0001); 15.38 ± 8.30 kg, t(84) = 16,868 (*p* < 0.0001); and 19 ± 9.06 kg, t(40) = 13,572 (*p* < 0.0001) at the 4th, 8th, 12th, and 24th weeks, respectively] (Figure 3). When we looked at the loss of weight as a percentage, we saw weight losses of 6.62 ± 2.1%, 10.75 ± 2.71%, 14.97 ± 6.81%, and 18.55 ± 4.63% at weeks 4, 8, 12, and 24, respectively (Figure 4). When we compared the initial BMI values with the values at 4, 8, 12, and 24 weeks, the values at follow-up were significantly lower (4th week: 35.77 ± 5.45 to 33.41 ± 5.26, t(568) = 53,538 (*p* < 0.0001); 8th week: 35.97 ± 5.71 to 32.10 ± 5.11, t(210) = 45,593 (*p* < 0.0001); 12th week: 37.26 ± 6.85 to 31.90 ± 5.69, t(84) = 27,814 (*p* < 0.0001); and 24th week: 37.66 ± 7.64 to 30.57 ± 5.23, t(40) = 15,283 (*p* < 0.0001)) (Figure 5). Considering how much the patients’ BMIs decreased compared to their initial values, BMI decreased to 2.36 ± 1.00 with t(568) = 53,538 (*p* < 0.0001); 3.88 ± 1.25 with t(210) = 45,593 (*p* < 0.0001); 5.36 ± 1.76 with t(84) = 27,814 (*p* < 0.0001); and 7.09 ± 2.93 with t(40) = 15,283 (*p* < 0.0001) at weeks 4, 8, 12, and 24, respectively (Figure 6).

**Figure 7:**
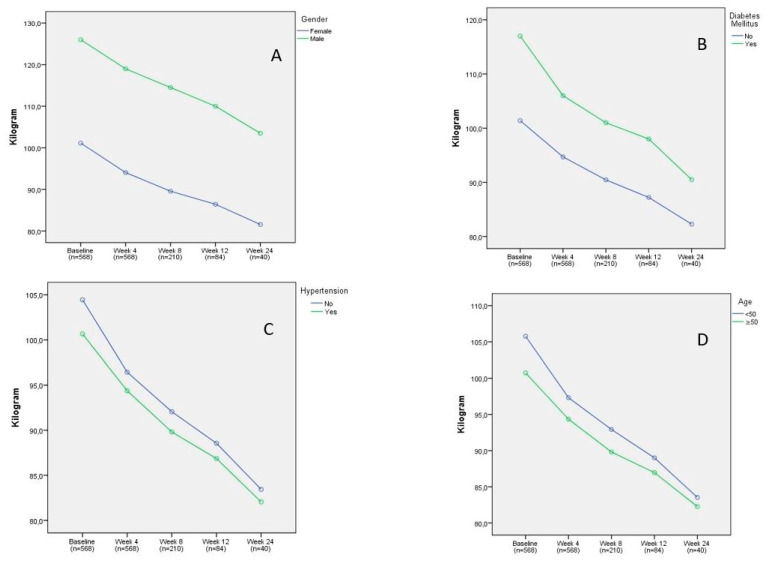
The impact of confounding factors on weight loss. (**A**) gender: *p* = 0.074, (**B**) diabetes mellitus: *p* = 0.451, (**C**) hypertension: *p* = 0.730, and (**D**) age: *p* = 0.673.

The impact of gender, diabetes, hypertension, and age on weight loss was not statistically significant (F = 2.951, *p* = 0.074; F = 0.581, *p* = 0.451; F = 0.121, *p* = 0.730; and F = 0.180, *p* = 0.673, respectively; Figure 7).

**Figure 8:**
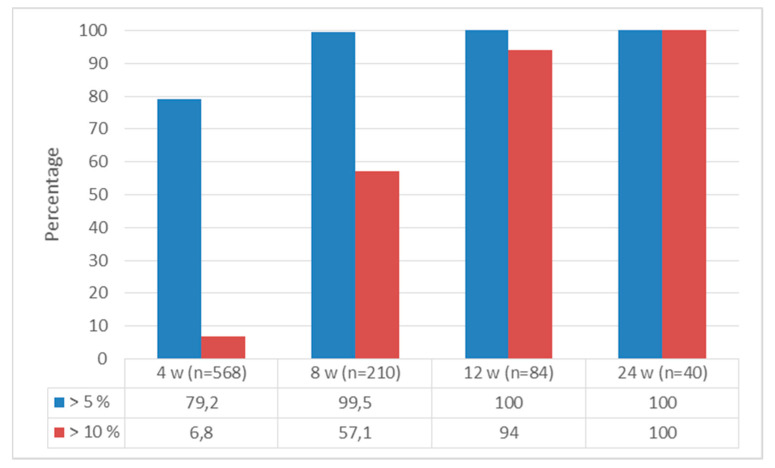
The proportion of patients who achieved weight loss of at least 5% and at least 10% of initial weight.

The percentages of patients who lost >5% and >10% of their initial weight were, respectively, 79.2% and 6% at the 4th week, 99.5% and 57.1% at the 8th week, 100.0% and 94.0% at the 12th week, and 100% and 100% at the 24th week (Figure 8).

During the follow-up period, the rate of patients who experienced at least one side effect was 70.01%; the most common side effects were nausea (51.58%) and heartburn GERD (4.22%), and no pancreatitis or hypoglycaemia was detected (Table 3).

## 5. Discussion

Although previous research has detailed the weight loss efficacy and side effects of liraglutide in the Turkish population, our study is the first of its size [17]. The recommended dosage of liraglutide for the management of weight is 3.0 mg. It is advised that treatment be initiated at 0.6 mg per day, with weekly increments up to the target dosage of 3.0 mg. Liraglutide exerts its anorectic effect by suppressing appetite, thereby reducing energy intake and promoting weight loss. In addition to dietary and exercise regimens, liraglutide is indicated for weight management in patients with a body mass index (BMI) of 27 or higher (in the presence of additional comorbidities) or a BMI of 30 or higher.

The medication is administered as a single daily dose at the same time each day, irrespective of meals. While liraglutide is generally well tolerated, the most commonly reported adverse effect is nausea. These symptoms are typically mild to moderate and do not necessitate treatment discontinuation. However, they are more prevalent during the titration period (4–8 weeks) and tend to subside with continued treatment [18]. The efficacy of the liraglutide molecule in promoting weight loss is contingent upon the dosage administered. In other words, the degree of weight loss increases in proportion to the dose administered. In the dose determination study, liraglutide was evaluated at doses of 1.2, 1.8, 2.4, and 3.0 mg, and it was observed that the weight loss provided by liraglutide increased in a dose-dependent manner. The highest degree of weight loss was observed in subjects administered a dose of 3.0 mg, with an average reduction in body weight of 7.3 kg [18]. A review of the literature reveals that the mean weight loss observed in clinical trials of liraglutide ranged from 4.1 ± 0.9 kg at 7 months in the Swiss study to 7.28 ± 6.2 kg at 6 months in the Canadian study, 10 ± 3.7 kg at 4 months in another Swiss study, and 7.4 ± 6.5 kg at 12 months in the Spanish study [19,20,21,22]. In the other study examining the Turkish population, the mean weight loss achieved with liraglutide at three months was 7.1 ± 4.01 kg [17]. In the course of our study, it was observed that the patients exhibited a greater degree of weight loss than what has been documented in the existing literature. At week four, the mean weight loss was 6.45 ± 2.32 kg, while at week eight, it reached 10.66 ± 3.41 kg. At week twelve, the mean weight loss was 15.38 ± 8.30 kg, and at week twenty-four, it reached 19 ± 9.06 kg. It is notable that the patients in this study exhibited a greater degree of weight loss compared to the findings of other studies.

In the liraglutide phase study, the mean BMI of the group receiving liraglutide was 36 ± 5.9 kg, with a change in BMI of 2.1 ± 2 in the group receiving treatment at 56 weeks [10]. The mean BMI at baseline was 40.8 ± 5.7 kg, while the mean BMI at four months was 37.5 ± 5.4 kg in the Swiss real-life study [19]. In our study, the mean BMI at baseline was 35.77 ± 5.45 kg/m^2^. At week 4, the BMI change was −2.36 ± 1.00 kg/m^2^ in 568 patients, aligning with the results obtained in the later weeks of other studies, even at this early stage.

FDA and EMA are currently evaluating three endpoints for the authorisation of anti-obesity drugs. The change in body weight was found to be 5% greater than that observed in the placebo group. Furthermore, the number of patients who experienced a weight loss of ≥5% was twice that observed in the placebo group. Moreover, a greater proportion of patients demonstrated a weight loss of >10% in comparison to the placebo group. Furthermore, numerous studies have demonstrated that patients must lose a minimum of 5% to 10% of their body weight to achieve a notable reduction in weight-related complications. The clinical development studies of liraglutide have also been designed with this objective in mind [10,11,18,23]. The primary endpoints of the liraglutide phase III clinical trial include weight change at week 56, the number of patients who experienced a weight loss of ≥5%, and the number of patients who experienced a weight loss of ≥10%.

At the conclusion of the study, a statistically significant reduction in body weight was observed in 92% of patients treated with liraglutide. The mean weight loss in patients using liraglutide was 8.4 kg (8%), with a particularly high success rate observed in patients who achieved ≥5% weight loss at week 12 with a treatment dose of 3.0 mg (early responder). In these patients, the mean weight loss at the conclusion of the treatment period was 11.5%. All values are statistically significant in favour of liraglutide; however, regarding the Swiss real-life data, this value was 4.2 ± 5% at seven months; in the Canadian study, it was 7.1 ± 5.4% at six months; in another Swiss real-life dataset, it was 8.7 ± 3.1% at four months; in the Korean study, it was 9.1 ± 3.6%; and in the previous study conducted in the Turkish population, it was 11.35 ± 5.22% at six months [10,17,19,20,22,24,25]. The mean decrease in weight as a percentage of initial body weight achieved in our study was 14.97 ± 6.81% at week twelve and 18.55 ± 4.63% at week twenty-four. In addition, in our study, as in the phase study, it was observed that patients who reached a weight loss target of 5% or more by week 24 had achieved weight loss by week 4.

A total of 63.2% of patients who were administered liraglutide achieved a weight loss of at least 5%. A total of 33.1% of patients treated with liraglutide achieved a weight loss of at least 10% in the phase study [10]. The studies incorporating real-world data revealed that, at seven months into the treatment period, 64.1% of patients in the Swiss study and 86% of those in the Korean study had lost at least 5% of their initial body weight. Furthermore, at 180 days, 34.5% of patients in the Swiss trial and 41.2% of those in the Korean study had achieved at least a 10% reduction in their body weight [24,26]. Similarly, significant weight loss has been reported in studies conducted in Spain and Canada [21,22]. In the other study, which examined the impact of liraglutide in the Turkish population, it was observed that 70% of patients achieved a weight loss of 5% or more at the three-month mark [17]. The findings of our study indicated that patients exhibited a high rate of weight loss even during the initial stages of the treatment programme. By the fourth week of treatment, 79.2% of patients had lost at least 5% of their body weight, while 6.8% had lost at least 10%. By the eighth week, these rates had risen to 99.5% and 57.1%, respectively, and the proportion of patients who achieved a weight loss target of more than 5% and 10% was, respectively, 100.0% and 94.0% at week twelve and 100.0% and 94.0% at week twenty-four. As with our other findings, it is notable that the proportion of patients who achieved weight loss of over 5% and 10% at the conclusion of the treatment period, which represents the primary endpoint of the phase studies, was higher than in the phase trials and other real-life studies examining Western populations.

Although liraglutide is generally well tolerated, the most commonly reported adverse effect is mild to moderate nausea [10,11,26]. This does not typically result in treatment discontinuation and is more prevalent during the initial titration period (4–8 weeks). With continued treatment, the incidence of nausea tends to diminish. As observed in the existing literature, nausea was the most prevalent adverse effect in our study, and no cases of pancreatitis were documented during the follow-up period.

Compared to phase studies and other studies with real-life data from Western populations, our study showed greater weight loss even in the early phase. The difference in weight loss outcomes may be attributed to the contrasting dietary habits between the populations; for instance, carbohydrate consumption is very high in Turkey. The patients included in the study were of a relatively high socioeconomic status and attended a private hospital, which may have resulted in a greater loss of weight compared to other studies. This is potentially due to their higher compliance with liraglutide treatment in terms of exercise and diet.

Although our study is the first of its kind to be conducted on this scale in the Turkish population, it is important to note that it was a retrospective study and that patient data were accessed from their files, which introduces two main limitations to the study. The absence of metabolic follow-up values for the patients and the relatively small number of patients included in the follow-up period must be acknowledged as potential limitations. In the Turkish healthcare system, the majority of applications are covered by the social security institution, thereby eliminating any financial burden on patients for examinations and laboratory tests. However, given that private hospitals levy charges for both examinations and laboratory tests, it may be reasonably inferred that the socioeconomic status of our patients is relatively elevated. Thus, the findings of our study cannot be extrapolated to the wider Turkish population.

## 6. Conclusions

Our study showed that liraglutide is an effective and safe treatment for weight loss in the Turkish population, consistent with the literature. In addition, unlike other real-world data, our study showed that significant weight loss was achieved even at early follow-up measurements such as at four and eight weeks. The selection of patients from private clinics in our study represents a significant limitation regarding the generalisability of the results to the Turkish population. Therefore, more comprehensive and prospective studies that are generalisable to the broader population are needed in this area.

## Figures and Tables

**Table 1:** Baseline characteristics of the study group.

	Mean ± SD (Min–Max)
**Age (year)**	42.37 ± 10.5 (18–71)
**Female sex, n (%)**	487 (85.6)
**Height (cm)**	165.51 ± 8.3 (142–197)
**Weight (kg)**	98.09 ± 17.48 (65–208)
**Body mass index (BMI) (kg/m^2^)**	35.77 ± 5.45 (27.05–72)
**Systolic pressure (mmHg)**	128.3 ± 19.13 (90–180)
**Diastolic pressure (mmHg)**	81.17 ± 11.55 (60–110)
**Total cholesterol (mg/dl)**	214.1 ± 41.15 (114–402)
**Low-density lipoprotein (mg/dL)**	139.08 ± 35.5 (15–279)
**High-density lipoprotein (mg/dL)**	54.06 ± 12.65 (18–103)
**Triglycerides (mg/dL)**	151.36 ± 91.70 (39–1133)
**Glucose (mg/dL)**	102.60 ± 25.388 (75–411)
**Aspartate transaminase (U/L)**	19.69 ± 10.2 (7–89)
**Alanine transaminase (U/L)**	26.95 ± 20.06 (2–188)
**Glycated haemoglobin (Hba1c) (%)**	5.69 ± 0.75 (4.4–12.7)

**Table 2:** Follow-up values of the study group.

	Week 4 (n = 568) Mean ± SD (Min–Max)	Week 8 (n = 210) Mean ± SD (Min–Max)	Week 12 (n = 84) Mean ± SD (Min–Max)	Week 24 (n = 40) Mean ± SD (Min–Max)
**Age (year)**	**42.37 ± 10.5 (18–71)**	**42.96 ± 10.98 (18–71)**	**44.58 ± 10.95 (19–67)**	**43.97 ± 10.57 (20–66)**
**Female sex, n (%)**	**486 (85.6)**	**188 (89.5)**	**77 (91.7)**	**38 (95)**
**Weight (kg)**	**91.63 ± 16.66 (59–193)**	**88.39 ± 15.92 (61–177)**	**85.90 ± 18.19 (64–169)**	**82.62 ± 15.95 (58–105)**
**Body mass index (BMI) (kg/m^2^)**	**33.41 ± 5.26 (24.6–66.8)**	**32.10 ± 5.11 (24.1–64.2)**	**31.90 ± 5.69 (24.4–58.5)**	**30.57 ± 5.23 (23.1–51.9)**
Diabetes mellitus, **n (%)**	**38 (6.7)**	**16 (7.6)**	**7 (8.3)**	**2 (5)**
**Prediabetes, n (%)**	**166 (29.2)**	**67 (31.9)**	**26 (31)**	**15 (37.5)**
**Hypertension, n (%)**	**369 (65)**	**125 (59.5)**	**45 (53.6)**	**22 (55)**

**Table 3:** Adverse effects of liraglutide during the follow-up period.

	n (%)
**None**	170 (29.99)
**Nausea**	293 (51.58)
**Constipation**	35 (6.16)
**Gastro-oesophageal reflux disease**	24 (4.22)
**Diarrhoea**	18 (3.16)
**Palpitation**	11 (1.93)
**Headache**	8 (1.41)
**Vomiting**	5 (0.80)
**Dizziness**	4 (0.7)

## Data Availability

The datasets used and analysed during the current study are available from the corresponding author upon reasonable request.
